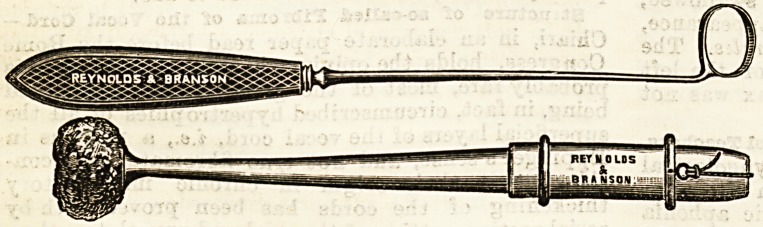# New Appliances and Things Medical

**Published:** 1895-10-26

**Authors:** 


### NEW APPLIANCES AND THINGS MEDICAL.

### NEW INSTRUMENTS FOR NA[illegible]O-PHARYNGEAL SURGERY.

(Reynolds and Branson, 13, Briggate, Leeds.)

Two new instruments of considerable practical use in naso-pharyngeal surgery have been manufactured by the above firm in accordance with the design of Mr. Bendelack Hewetson, of Leeds. The first of them, a new sponge holder, made either in celluloid or vulcanite, is provided with a hollow central stem, through which passes a piece of whip-cord, fastened firmly at one end to the sponge, while the other end is made fast to a ring which is slipped over the end of the handle. The sponge is thus held tightly in the trumpet-shaped orifice of the holder. The advantages claimed for this instrument are: (1) The ease with which it can be cleaned and kept clean; (2) the avoidance of injury to the soft parts, which sometimes follows the employment of metal holders; (3) the durability and reliability of the instrument; it cannot be bent or destroyed by ordinary use. The price is 7s. 6d. in celluloid, and 3s. 6d. in vulcanite. The second instrument referred to is a new form of scraper for the removal of adenoid vegetations in the naso-pharynx or pharynx. The instrument is in form something like the ordinary scraper, but the ring is semi-circular, with a double scraping edge, which can by easy manipulation operate in an upward, backward, or downward direction, and even effect the removal of adenoids which occupy the pharynx proper. It is claimed that adenoids in any position can be more readily removed by this instrument than by the older stirrup-shaped ring, and further, its range of usefulness is considerably more extensive, since parts inaccessible to the older form can be readily reached by simple manipulation of the cutting edges.

## Figures and Tables

**Figure f1:**